# Medicine and Doctors

**Published:** 1872-10

**Authors:** 


### The Bistoury.

ELMIRA, N. Y., OCT., 1872.

The BISTOURY is published Quarterly, upon the 1st of April, July, October and January, at Fifty Cents a year, in advance.

### MEDICINE AND DOCTORS.

The habit of prescribing for the sick may be said to be universal. Every country and clime has its doctors or medicine men. The confidence reposed in practitioners of the healing art, by their patrons, is certainly worthy of the marvelous results ascribed to their efforts. The haste with which the family physician is summoned to the bed-side of a sick member of the household, is only equalled by the alacrity with which a nauseous potion is prescribed by the former and swallowed by the latter. A physician who does not prescribe medicine, even when he plainly sees it is not indicated, is considered by anxious friends and the patient himself, a very useless personage in the sick chamber. People are so given to the use of drugs, and have such implicit faith in their efficacy, that it is as much as the reputation of the physician is worth to attempt to convince them that medicine is rarely needed, and always overestimated, in the cure of the ordinary ailments of the family. Should the physician be called to a case of scarlet fever, and spend a few moments directing the mother how to proceed with the child, advising the administration of little pellets of ice frequently during the day, and the application of ice-bags to the throat of the little sufferer, asking her not to keep the patient in bed, nor yet within doors, (if it preferred to be up and about), and after some directions with reference to bathing and diet, should take his hat and depart, without leaving a dose of medicine, he would more than likely be considered a lunatic by that mother, who has always been accustomed to administer hot drinks "to keep the fever out," and numerous powders and potions to be taken at stated intervals, the effects to be accomplished by which she was entirely ignorant of, as was also the physician.

There exists a law in nature, known to physicians as *vis medicatrix naturæ*, the tendency of which is to restore that which has been destroyed through accident or disease. This is illustrated in the limb of a tree, which, if partly broken, is restored; should the loss be complete, a new growth is speedily seen, and the original conformation of the tree restored. Animals, it has been found, are subjected to many of the diseases common to man. They never take medicine, yet are their recoveries more frequent than in our race. Many of the diseases common to the human family, are self-limiting, that is, if left entirely alone, without medication, will subside within a limited number of days, in obedience to certain mysterious laws, governing them. We might give as examples, measles, scarlet fever, small-pox, etc.—These diseases, if allowed to run without medication, will, under favorable nursing, terminate by limitation in a certain number of days, common to each. We do not declare that *all* cases of the above mentioned diseases will recover without medication, neither will they with it, but we accept the authority of the best intellect of our profession, who affirm that little or no medicine is necessary in such cases, and that those who receive no medicine make the best recoveries.

If medicines are as effective in arresting disease as we are fond of supposing, why is it that we are unable to name the best educated and most skillful physician in every community, by the results of his practice? Certainly, he who is best versed in the theory and practice of medicine, and in the diagnosis of disease, should show the best record in curing suffering humanity! While we have no difficulty in recognizing a highly educated physician from a charlatan, by his scholarly attainment and gentlemanly deportment, yet would we be unable to select him from the latter by his success from the administration of drugs. This is a lamentable and notorious fact, and should be of sufficient consequence to shake the faith of every intelligent observer, in the efficacy of medicines in disease.

The most impudent and ignorant quack never lacks for patrons—he will always find some one equally ignorant with himself, willing and anxious to swallow his pill. Even people whom we are in the habit of accrediting with considerable intelligence, will allow themselves to be duped by the most wretched of creatures, and swallow doses, which, if prescribed by an intelligent physician, would subject him to a suit for mal-practice.

We have known people who pass for intelligent beings, and of whom we would expect more discrimination, to gulp the most vile decoctions, and follow it with hourly doses of physic, until faintness or unconsciousness only prevented the possibility of further dosing, and all patiently taken because an ignorant old woman, by some unaccountable means, induced them to believe that she was in hourly communication with the spirit of some defunct, old doctor, who prescribed such a course of treatment, and who could see directly in, and through them, locating tape-worms in their stomachs (?), and worms, eels and various other creeping things in their livers, hearts and lungs! Hundreds of these people take gin flavored with rheubarb, "boneset," or egg, daily, and pronounce themselves better, yea, better than for years before.

Another fact, calculated to stagger the faith of the most enthusiastic believer in the virtue of medication, is that of the existence of two flourishing schools of medicine, one entirely antagonistic to the other, yet each claiming among its respective adherents, equally intelligent and enthusiastic people.

The one class calmly submit to cupping, leeching, bleeding, blistering, purging, and the administration of large and nauseous doses of marvelously and mysteriously compounded potions, and firmly believe they are rendered better thereby; while the opposing party take little sugar pellets, in large doses of water, and minus the suffering from blisters, etc., make equally as rapid recoveries, and are accordingly as dutifully thankful. Both cannot be right, yet, both declare their cures, and the fact cannot be gainsayed—both parties recover!

From this we argue that the efficacy of drugs in the healing of disease, has been greatly overestimated, and that many of the so-called cures ascribed to physicians and their medicines—either in large or infinitesimal doses—are directly attributable to that great and grand law of cure, *vis medicatrix naturæ*.

We do not wish to be understood as declaring that medicine is never necessary or useful in combating disease—not at all, but we do insist that too much medicine is given, and that it is administered when not needed, to the great harm of the patient. If people would only discriminate between intelligent, educated physicians, and the abominable humbugs and numbskulls that infest every community, and if they would learn to appreciate the advice and counsel of their physicians, and not open their mouths to swallow a pill every time he enters their door, they will have made an advance that will redound greatly to their health and credit.

